# Unfolding the Mild to Moderate Short-Term Side Effects of Four COVID-19 Vaccines Used in Bahrain: A Cross-Sectional Study

**DOI:** 10.3390/vaccines9111369

**Published:** 2021-11-22

**Authors:** Muhammad Nauman Zahid

**Affiliations:** Department of Biology, College of Science, University of Bahrain, Sakhir 32038, Bahrain; nzahid@uob.edu.bh; Tel.: +973-3320-5242

**Keywords:** COVID-19, vaccines, side effects, Pfizer, AstraZeneca, Sinopharm, Sputnik

## Abstract

Severe Acute Respiratory Syndrome Coronavirus 2 (SARS-CoV-2) created a global pandemic (COVID-19) that has resulted in massive health and economic losses. The current unavailability of treatments leaves vaccination as the only way to control this disease. There are four vaccines (Sinopharm, Pfizer—BioNTech, Sputnik, and AstraZeneca) available in Bahrain. This project aimed to study the most common side effects resulting from the first and second doses of these four vaccines. Data were collected through an online questionnaire answered by 311 individuals who received both doses of one of these four vaccines. The results of this study revealed that regardless of the vaccine identity, participants experienced more side effects from the second dose. Among the different side effects, pain at the site of injection was primarily observed after the first dose of the Pfizer vaccine (43%), which was followed by the AstraZeneca vaccine (31%). Moreover, fever was observed in participants after the first dose of the Sputnik vaccine (37%), while headache was mainly observed after the first dose of the Pfizer vaccine (32%). It is important to note that fatigue was observed after the first dose of all four vaccines but was reported by the highest proportion of respondents in the Pfizer group (28%). Interestingly, there are some side effects, such as pain at the site of injection, that are correlated with fever (*r* = 0.909). Similarly, headache is correlated with fever (*r* = 0.801) and pain at the site of injection (*r* = 0.868). Overall, it was observed that recipients of the Sinopharm vaccine reported the mildest side effects among all four vaccines. The crucial finding of this study is that the first and second dosage post-vaccination side effects were modest and predictable with no occurrences of hospitalization; this information can assist in lessening vaccine apprehension.

## 1. Introduction

The coronavirus disease 2019 (COVID-19) first reported in December in Wuhan, China, as an epidemic [1]. On 11 March 2020, the World Health Organization (WHO) declared COVID-19 a worldwide pandemic [2]. The number of confirmed cases of COVID-19 continues to rise every day. Many studies have been conducted to clarify the epidemiology, virology, and clinical management of SARS-CoV-2. Researchers have not yet identified or approved any drugs against SARS-CoV-2 due to a lack of sufficient data and understanding of the virus [1]. Early studies showed that one infected person spread the disease to two or more people, which leads to a high spread rate [3,4,5,6]. Symptoms of COVID-19 include shortness of breath, diarrhea, abdominal pain, chest pain, and loss of smell and taste [7,8,9]. In some cases, infection can lead to dysfunction of the kidneys, lungs, heart, and in severe cases, even death. Diagnosis is performed by RT-PCR assay using specimens collected from oropharyngeal or nasopharyngeal swab [10]. 

It has been reported that 11,503 clinical studies and 2388 randomized controlled trials (RCTs) were registered for COVID-19 [11]; there is not yet an antiviral treatment that is efficient in all patient populations. Thus, there is an immediate need to vaccinate the entire population against the SARS-CoV-2 virus, which is thought to be the most efficient means of ending this pandemic. Before the COVID-19 pandemic, there was a well-established body of information detailing the structure and role of coronaviruses that cause diseases such as SARS and MERS, allowing for the rapid development of various vaccine technologies in early 2020. SARS-CoV-2 genetic sequence data were shared via the Global Influenza Surveillance and Response System (GISRS) on 10 January 2020, and the global pharmaceutical industry announced a significant commitment to addressing COVID-19 on 19 March 2020 [12]. 

There were 308 vaccine candidates in different stages of development as of March 2021, with 73 in clinical trials, including 24 in Phase I trials, 33 in Phase I–II trials, and 16 in Phase III development [10]. Several COVID-19 vaccines have shown efficacy in preventing symptomatic COVID-19 infections in Phase III trials. These include two RNA vaccines (Pfizer–BioNTech vaccine and Moderna vaccine), four traditional inactivated vaccines (BBIBP-CorV, CoronaVac, Covaxin, and CoviVac), four viral vector vaccines (Sputnik V, AstraZeneca vaccine, Convidecia, and the Johnson & Johnson vaccine), and two protein subunit vaccines (EpiVacCorona and RBD-Dimer) [13].

The development of these vaccines generated hope for an end to the COVID-19 pandemic. However, many were afraid of taking these vaccines due to myths, misconceptions, and side effects. Many studies have reported the unique side effects of individual vaccines [14,15,16,17], but few studies discussed the side effects of different vaccines [15,18]. 

Common side effects of the above-mentioned vaccines are pain at the site of infection, fever, headache, fatigue, joint pains, cough, myalgia, and nausea [14,17]. Following COVID-19 immunization, two self-limiting symptoms, myocarditis and pericarditis, were reported. In younger patients, myocarditis developed quickly, usually after the second vaccine. After either the first or second dose, older patients developed pericarditis [19]. COVID-19 vaccines have been linked to an increase in venous thromboembolic (VTE) events, which was likely due to a thrombophilic state caused by inflammation and immune thrombosis [20]. Due to reports of thromboembolic events in vaccinated patients, distribution of the AstraZeneca vaccine was suspended in many European countries by mid-March 2021. By 10 March 2021, the European Medicines Agency (EMA) had received 30 reports of thromboembolic events (primarily venous) [21]. Despite these reports, the European Medicines Agency (EMA) ruled on 18 March 2021 regarding the Oxford–AstraZeneca COVID-19 vaccination that “benefits currently exceed the risks notwithstanding a probable association to uncommon blood clots with low blood platelets [22]. 

There is little published data to corroborate adverse responses to the COVID-19 vaccination. Only a few studies discuss the side effects of the Sinopharm vaccines [15,18,23,24,25]. The most common adverse effects of Sinopharm vaccination, according to these studies, were weariness, injection site pain, headache, fever, lethargy, chills, and myalgia. Some studies reported the side effects of the Pfizer vaccine [26,27,28,29,30,31,32,33], although the findings support no unusual patterns of adverse events following two doses of the COVID-19 vaccine; the majority of these reactions were mild to moderate. Studies describing the side effects of the AstraZeneca vaccine are also limited, with reported symptoms similar in type and severity to those of the Pfizer vaccine [29,30,31,34,35]. Although there are worries regarding the safety of the AstraZeneca vaccine following instances of blood clots, it has been observed that post-vaccination neurological problems are uncommon [30]. The first or second dose of the Sputnik vaccine has been reported to cause mild to severe adverse effects that generally resolve in 0–3 days, although investigations into the side effects of this vaccine are limited [34,36,37,38].

The Kingdom of Bahrain has approved only four vaccines: the Pfizer-BioNTech vaccine, Sputnik V, the AstraZeneca vaccine, and the Sinopharm vaccine. However, vaccine hesitancy remains high due to concerns regarding vaccine safety, side effects, and effectiveness against the COVID-19 virus. Therefore, this study was designed to determine the side effects of the COVID-19 vaccines provided by the Ministry of Health in the Kingdom of Bahrain. 

## 2. Materials and Methods

### 2.1. Study Design and Participants

A randomized, cross-sectional study was conducted from 10 April to 15 May 2021, on Bahraini people who were vaccinated against COVID-19. Citizenship, ethnicity, occupation, and location of residence were not considered. Adults (18 years and older) were invited to participate in a self-administered online survey (Google Form) that was distributed through social media platforms (e.g., Facebook, Twitter, etc.). Potential participants were first informed about the study through distribution of a detailed description of the study’s purpose. Then, those who wished to participate were asked to sign a mandatory electronic informed consent form that included statements regarding voluntary participation and anonymity. The participants who took only one shot of vaccine were not included in this study. Three hundred and eleven participants were included in this study, while 24 participants were excluded, as they took only 1 dose of any vaccine. This study was approved by the ethics committee of the Department of Biology. 

### 2.2. Questionnaire (Survey)

A questionnaire was prepared following a thorough review of the literature on databases including but not limited to Google Scholar and PubMed. The participants were informed in the questionnaire about some medical terms, for example, anosmia (loss of smell), dysgeusia (loss of taste), myalgia (muscle pain), and dyspnea (shortness of breath). This extensive literature review revealed a wide range of potential post-vaccination side effects that were subsequently considered in the design of the survey. Several questions requested demographic information, participants’ general health status vaccination, and pre-and post-vaccination imprints of COVID-19 vaccinations. The survey was developed in English and modified following review by a panel of experts. The poll was translated into Arabic before being testing and distribution.

A pilot study was conducted with 40 participants to test the clarity and comprehensibility of survey material. These pilot study participants were not included in the formal evaluation. Survey reliability was determined using the Cronbach’s alpha test of internal consistency. In this study, the test indicated that the survey instrument was generally reliable, with a Cronbach’s alpha of 0.79.

### 2.3. Data Analysis

The data were categorized based on the type of vaccine and side effects following vaccination, such as headache, fatigue, myalgia, fever, nausea, and injection site pain. The data were stored in Google forms until final responses were downloaded and used for data analysis. Univariate models were applied to estimate the percentage of symptoms reported after receipt. Pearson’s correlation was applied to understand the association between each pair of symptoms. The null hypothesis for the Shapiro–Wilk test confirmed that the data were normally distributed in this sample population. 

## 3. Results

Table 1 shows the characteristics of participants who received both doses of any of the four vaccines available in the Kingdom of Bahrain. The respondent cohort was 56.3% male and 43.7% female. There were four age categories: 22% of participants were 18–24 years, 29.9% were 25–34 years, 34.7% were 35–44 years, and 13.1% of participants were 45 years of age or above. In total, 16.7% of respondents were single, 75.2% were married, and 8.1% were divorced or widowed. The majority, 70.7%, were employed, 21.2% were students, and 8% were unemployed. Just over half (52.1%) were Bahraini, while 47.9% were non-Bahraini. Most (81%) individuals were healthy, while 19% reported a chronic illness. According to Table 1, the most common chronic conditions were diabetes (8%) and cardiovascular diseases (5.4%). 

**Figure 1:**
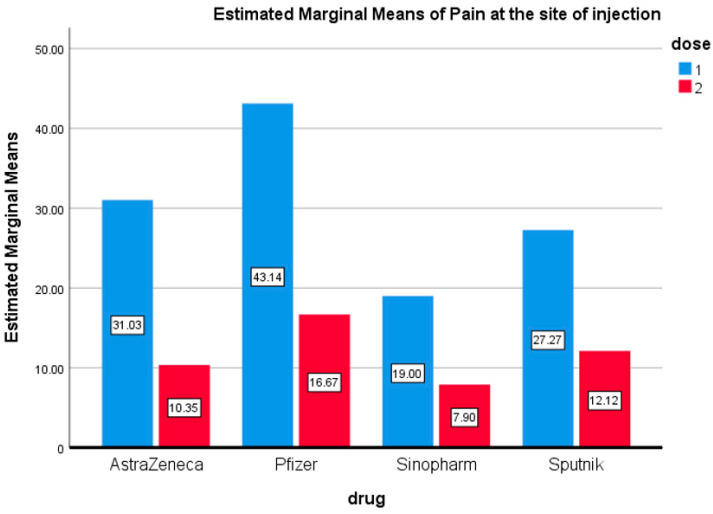
Pain at the Injection Site. Estimated marginal means of pain at the site of injection after the first and second doses of vaccines used in the Kingdom of Bahrain.

The number of participants was standardized for each vaccine to study the side effects of each vaccine using ANOVA. Figure 1 shows that the greatest proportion of respondents who reported pain at the site of injection after the first dose received the Pfizer vaccine (43.14%), followed by the AstraZeneca vaccine (31.03%). The lowest proportion of pain complaints was reported by those who received the first dose of Sinopharm (19%). However, this difference in reports of pain after the first dose among the vaccine groups was not statistically significant. Interestingly, the proportion of respondents who complained of pain at the site of injection after the second dose was reduced for all vaccine groups. The difference in reports of pain at the injection site between the first and second dose for all four vaccines is statistically significant (0.012) (Figure 1). 

**Figure 2:**
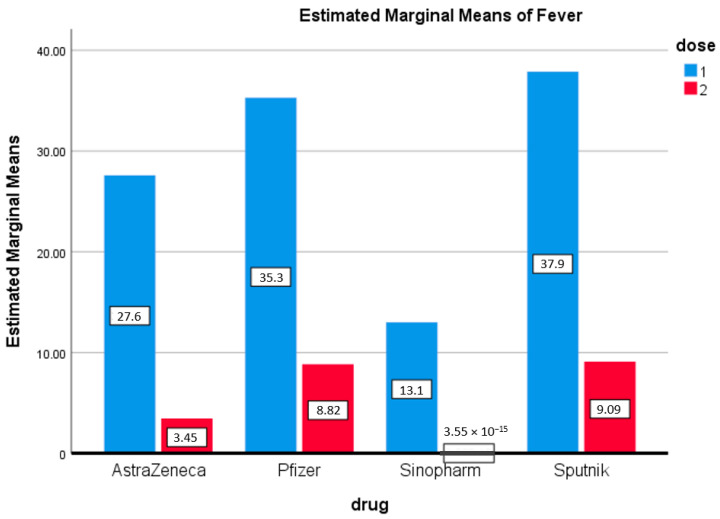
Fever. Estimated marginal means of fever after first and second doses of vaccines used in the Kingdom of Bahrain.

Figure 2 shows that most respondents who suffered from fever after the first dose of vaccination received Sputnik (37%) or Pfizer (35.3%). Very few participants suffered from fever after taking the first dose of the Sinopharm vaccine (13%). It is important to note that the second dose of all four vaccines had a mild effect on individuals, and there is a statistically significant difference in symptoms of fever after the first and second doses for all four vaccines (0.007) (Figure 2).

**Figure 3:**
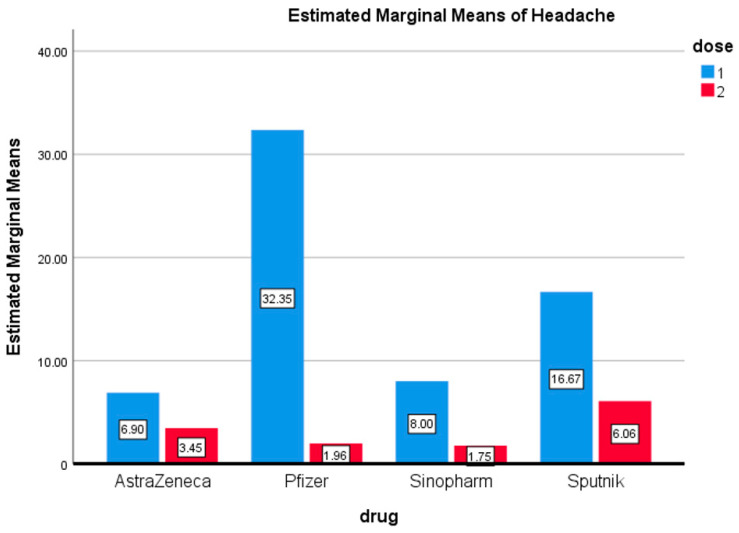
Headache. Estimated marginal means of headache after first and second doses of vaccines used in the Kingdom of Bahrain.

Another critical side effect that was observed after both doses was headache. The highest proportion of individuals reported headaches after the first dose of Pfizer (32.35%), which was followed by Sputnik (16.67%), whereas only 6% of participants felt headaches after the first dose of AstraZeneca and 8% after Sinopharm. Similar to the previously mentioned side effects, respondents reported less severe symptoms compared to the first dose. It was noticed that the second dose of Sputnik caused a headache in more participants (6.06%) than the other three vaccines (Figure 3). The difference in the marginal mean of headache among the first and second doses for these four vaccines was not statistically significant.

**Figure 4:**
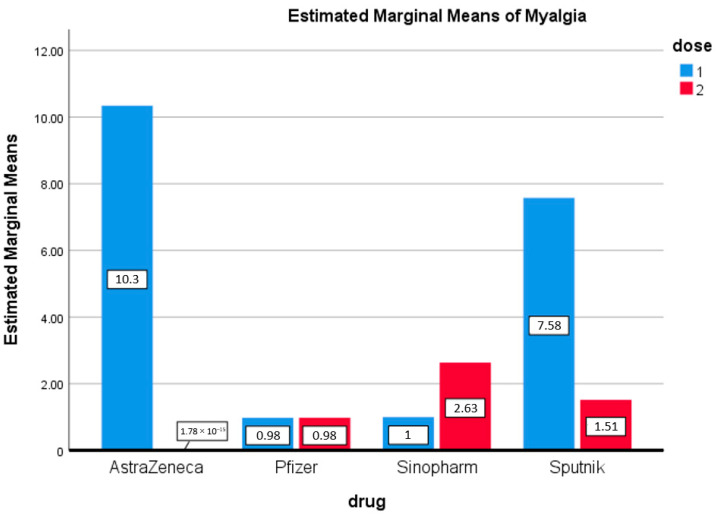
Myalgia. Estimated marginal means of myalgia after first and second doses of vaccines used in the Kingdom of Bahrain.

Few participants complained of myalgia after either the first or second doses of any of the four vaccines. A small number of participants reported myalgia after the first dose of AstraZeneca (10.3%) and Sputnik vaccinations (7.5%) (Figure 4).

**Figure 5:**
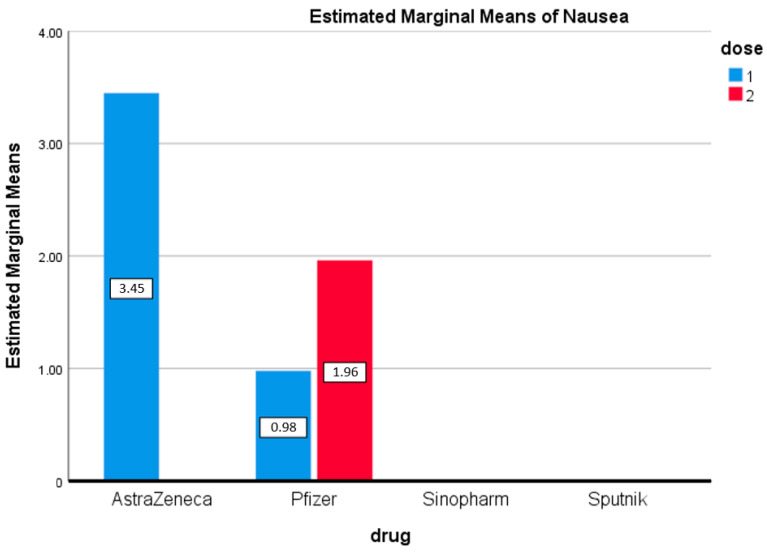
Nausea. Estimated marginal means of nausea after first and second doses of vaccines used in the Kingdom of Bahrain.

It is interesting to note that nausea was observed in participants who received the first doses of AstraZeneca (3.45%) and Pfizer (0.8%), but in the Pfizer group, nausea was observed only after the second dose (1.96%) (Figure 5). The difference in the estimated marginal mean between the first and second doses of Pfizer (0.575) was not statistically significant. 

**Figure 6:**
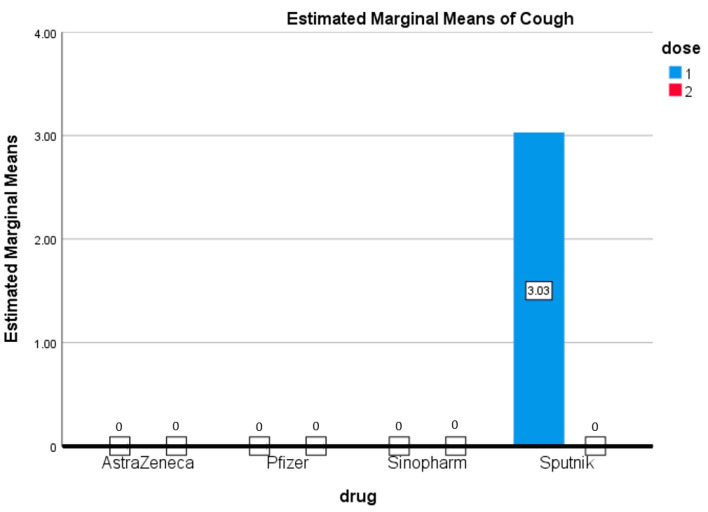
Cough. Estimated marginal means of cough after first and second doses of vaccines used in the Kingdom of Bahrain.

Figure 6 shows that the only participants who suffered from cough received the first dose of the Sputnik vaccine (3.03%). People who received the other three vaccines did not suffer from cough after either the first or second dose.

**Figure 7:**
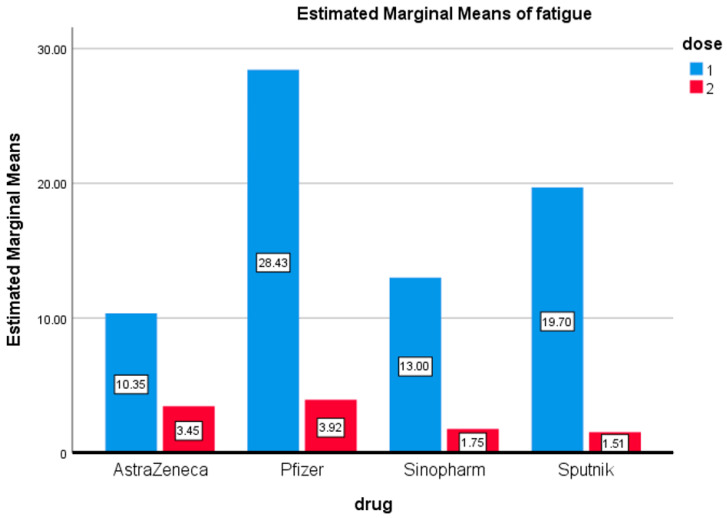
Fatigue. Estimated marginal means of fatigue after first and second doses of vaccines used in the Kingdom of Bahrain.

Fatigue was one of the most common symptoms reported after COVID-19 vaccination. Following the first dose, fatigue was reported primarily by those who received Pfizer (28%), followed by Sputnik (20%), then Sinopharm (13%). Notably, very few individuals reported fatigue as a side effect after receiving the second dose of any of the four vaccines (Figure 7). The difference in fatigue after the first and second doses of these vaccines was statistically significant. 

**Figure 8:**
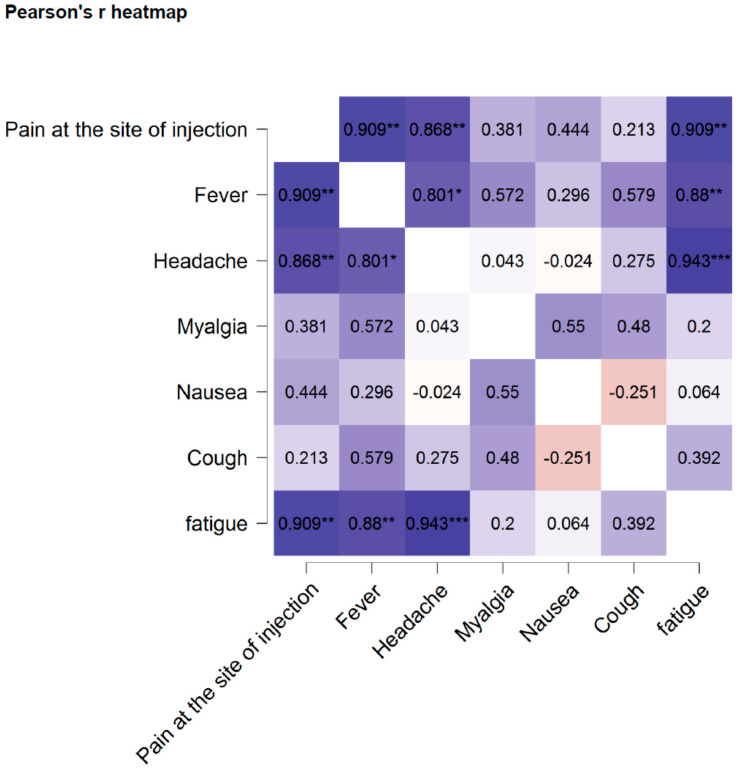
Pearson’s heatmap. Pearson’s heatmap illustrates the correlation among different side effects of COVID-19 vaccines that have been used in the Kingdom of Bahrain. * *p* < 0.05, ** *p* < 0.01, *** *p* < 0.001.

Pearson’s heatmap was applied to determine the correlation of reported side effects of four COVID-19 vaccines that have been used in the Kingdom of Bahrain. As shown in Figure 8, some side effects, such as pain at the site of injection, are correlated with fever (*r* = 0.909). Headache is also correlated with fever (*r* = 0.801) and pain at the site of injection (*r* = 0.868). Some symptoms, primarily myalgia and cough, seem to be independent of any other side effect. 

## 4. Discussion

This study analyzed the side effects of first and second doses of four vaccines that have been used in the Kingdom of Bahrain. The results of this study revealed that the first dose of all four vaccines had more side effects than the second dose of these vaccines. Among different side effects, pain at the site of injection primarily observed after the first dose of the Pfizer vaccine (43.14%) followed by the AstraZeneca vaccine (31.03%). Fever was observed in the highest proportion of respondents who following the first dose of the Sputnik vaccine, while headache was mainly observed after the first dose of the Pfizer vaccine (37%). It is important to note that fatigue was observed after the first dose of all vaccines, most predominantly in individuals who received the Pfizer vaccine (28%). Surprisingly a few side effects are correlated to each other: pain at the site of injection and fever (*r* = 0.909), headache and fever (*r* = 0.801), and headache and pain at the site of injection (*r* = 0.868) In contrast nausea, myalgia, and cough are not associated with other side effects. 

The topic of this study is crucial due to the prevalence of vaccine hesitancy. It is a time to provide the public with data on the side effects of these vaccines. A few studies have reported on the side effects of a particular vaccine. Minimal reports are available where scientists have compared data on the side effects of four vaccines. The comparative nature of this study will play a key role in alleviating fears about the side effects of COVID-19 vaccines. A comparison of few important studies has been discussed in the Table 2.

Recently reports have highlighted the side effects of the Sinopharm vaccine [24]. These studies reported that normal injection site pain (42.2%), fatigue (12%), fatigue (12.2%), and headache (9.6%) were the most prevalent adverse effects following the first dose. Interestingly, the current study reported similar results of fever (13%), fatigue (13%), and headache (8%) after the first dose of Sinopharm (Figure 1, Figure 2 and Figure 3 and Figure 7). Abu-Halaweh and colleagues [25] studied the side effects of the Sinopharm and Pfizer vaccines. They reported that 46.3% of individuals experienced an adverse response after the first dose, with injection site pain as the most common complaint (33.2 percent). The second dose caused adverse effects in 48.6% of individuals, with injection site pain as the most common complaint (29%). All these studies, including this current study, reported that the most prevalent adverse effects of the Sinopharm vaccine were fatigue, injection site pain, headache, laziness, chills, myalgia, and fever.

Several studies have reported the side effects of the Pfizer vaccine [23,26,27,28,29,30]. Shitany and colleagues [26] reported that pain at the injection site, headaches, flu-like symptoms, fever, and fatigue were the most prevalent complaints following the Pfizer vaccine. In this study, it was observed that after the first dose of Pfizer, 43% of people experienced pain at the site of injection, 25% suffered from fever, 32% developed a headache, while 28% reported experienced fatigue. Riad and colleagues [27] conducted a cross-sectional survey in which they found that 89% of people experienced injection site pain, 62.2% experienced fatigue, 46% suffered from headache, 37% felt muscle pain, and 34% experienced chills. In another study [28], the authors described that 54.8% of people experienced more side effects following the second dosage of the Pfizer vaccine than the first with 15.8% of individuals reporting fewer side effects after the first dose. The CDC team found that the side effects of the additional (booster) dose were mild to moderate and were identical to those observed after receiving the first two doses [32]. Only the Pfizer vaccine is currently approved for use in the pediatric population aged 16 and up under emergency use authorization [33].

AstraZeneca is the third Bahrain-approved vaccine developed to combat COVID-19. Interestingly, a study reported the short-term side effects of Pfizer and AstraZeneca in Saudi Arabia [29]. Similar to the previously mentioned studies, 60% of trial participants reported side effects from COVID-19 vaccinations, with the majority reporting fatigue and pain at the injection site. Concerns have been raised about the safety of the AstraZeneca vaccine due to reports of blood clots, but post-vaccination neurological events are currently rare [30]. Menni and colleagues studied the side effects of Pfizer and AstraZeneca in the United Kingdom [31]. They found that 7% of recipients experienced headache after the first dose of Pfizer while 22% developed headache after the first dose of AstraZeneca. Another study found that the AstraZeneca vaccine caused non-life-threatening side effects [35]. Denis Logonov and colleagues published interim results from a Phase III study of the Sputnik V COVID-19 vaccine. They demonstrated a robust protective impact across all age categories of participants [36]. It has been reported that the first or second dosage of the Sputnik vaccine confers mild to severe side effects that usually disappear within three days [34]. Nogrady and his colleagues reported that the Sputnik vaccine is not only safe but effective against SARS-CoV-2 [37]. These studies are in agreement with our results that the side effects of the Pfizer, AstraZeneca, Sputnik, and Sinopharm are mild to moderate, and they remain for only a few days. 

The primarily non-life-threatening short-term side effects reported in this study could help to dispel conspiracy theories and reassure vaccine-apprehensive members of the public of the safety of this vaccine. Nevertheless, there are few limitations of such self-reported online surveys. The reliance of our results on this method could have resulted in information bias due to misclassification of side effects. Furthermore, the sample diversity may differ from the wider public cohort due to the sole inclusion of people with internet access. The sample was not evenly distributed across gender or career, necessitating caution in generalizing the findings. However, these data are instrumental for the vaccine campaign, which is the only solution in the fight against the COVID-19 pandemic. 

## 5. Conclusions

In conclusion, all four vaccines available in Bahrain have short-term side effects that are modest in frequency, mild in intensity, and short-lived. These findings may help to boost public trust in the safety of COVID-19 vaccinations, hastening the immunization process in Bahrain by dispelling misconceptions and conspiracy theories concerning post-vaccination side effects.

## Figures and Tables

**Table 1:** Characteristics of participants who received both doses of different vaccines.

Variable	Participants	AstraZeneca	Pfizer	Sinopharm	Sputnik
Gender	Male	18	55	63	39
	Female	11	47	51	27
Age	18–24	2	23	24	20
	25–34	10	29	40	14
	35–44	12	38	36	22
	45+	5	12	14	10
Marital Status	Single	03	17	20	12
	Married	24	74	87	49
	Divorced	02	11	07	05
Nationality	Bahraini	13	54	60	35
	Non-Bahraini	16	48	54	31
Employment status	Employed	22	77	81	40
	Student	4	21	22	19
	Unemployed	3	4	11	7
Pre-existing medical conditions	Healthy	26	84	97	47
	Cardiovascular	-	6	4	3
	Diabetes	1	7	7	10
	Cholesterol	1	3	5	4
	Diabetes and cholesterol	1	2	1	2

**Table 2:** Comparison of different studies showing side effects of COVID-19 vaccines.

Author	Vaccine under Study	Country of Study	Total Participants/No. of Participants Included in Study	Age	Sex Male/Female	Risk of Bias/Limitations
Saeed et al., 2021	Sinopharm	UAE	1102/1080	≥18	320/760	70.3% females in their study, so there may be risk of bias.
Abu-Halaweh et al., 2021	Sinopharm, Pfizer	Jordan	1004/1004	≥18	673/331	observational study based on semi-structured interviews conducted over the phone. There is no standard method to report the severity of the side effects.
Hatmal et al., 2021	Sinopharm, AstraZeneca, Pfizer-BioNTech,	Jordan	2237/2237	≥18	869/1344	self-reported online survey, misclassification of results, gender and profession were not evenly distributed in the sample.
Riad et al., 2021	Pfizer	Czech Republic	922/887	≥18	100/776	The occurrence of side effects was unrelated to first or second dose, samples were not equally distributed across gender or profession.
Alhazmi et al., 2021	Pfizer, AstraZeneca	Saudi Arabia	535/515	≥18	221/294	self-reported survey, distribution was dependent on author’s convenience, most of the participants were young
